# Precision diagnostics in bronchiectasis: current advances in imaging, microbiology, biomarkers, and digital health

**DOI:** 10.3389/fmed.2026.1907636

**Published:** 2026-07-30

**Authors:** Bowen Wu, Sha Lu, Haipeng Liu

**Affiliations:** Department of Respiratory Medicine, Xiangzhou District People's Hospital, Zhuhai, China

**Keywords:** artificial intelligence, biomarkers, bronchiectasis, digital health, metagenomic next-generation sequencing, microbiome, precision medicine, quantitative CT

## Abstract

Bronchiectasis is a complex, chronic airway syndrome driven by a vicious cycle of irreversible bronchial dilatation, impaired mucociliary clearance, recurrent infection, and tissue-destructive inflammation. Reflecting its profound clinical heterogeneity, patients with identical structural damage on high-resolution computed tomography (HRCT) often exhibit divergent profiles in airway microbiology, inflammatory endotypes, exacerbation frequencies, and therapeutic responses, indicating that static anatomical classification fails to capture disease complexity. Sole reliance on visual CT inspection, standard sputum cultures, and subjective symptom tracking misses the driving mechanisms of individual disease progression. Emerging modalities—artificial intelligence (AI)-driven quantitative imaging, molecular microbiology, high-throughput biomarker profiling, and digital remote monitoring—aim to address these gaps. Our analysis shows that while these tools cannot substitute for bedside clinical acumen, they clarify obscure phenotypes, expose actionable treatable traits, and enable earlier, preemptive strategies. This review evaluates these contemporary diagnostic frameworks in non-cystic fibrosis bronchiectasis, dissecting their clinical utility, evidentiary maturity, and the economic and logistical barriers to routine adoption. Given that current evidence remains fragmented, advancing the field demands standardized imaging protocols, transparent algorithmic pipelines, clinically actionable metagenomic reporting, and robust validation in underrepresented Asian and Chinese cohorts. The real challenge lies not in generating more data, but in integrating these heterogeneous, high-dimensional datasets into pragmatic, point-of-care decision pathways that improve patient outcomes without widening disparities in global healthcare delivery.

## 1 Introduction

Bronchiectasis presents as a heterogeneous, chronic clinical syndrome rather than a monolithic disease entity. Radiologically defined by abnormal and usually irreversible bronchial dilatation, its clinical course reflects a destructive interplay among impaired mucociliary clearance, microbial colonization, persistent airway inflammation, structural tissue injury, and host susceptibility (1, 2).

The classic “vicious cycle” model provides a useful conceptual baseline. However, individual patient trajectories diverge upon encountering this pathology. While certain individuals exhibit profiles dominated by intense neutrophilic inflammation and chronic bacterial infection, others feature allergic, eosinophilic, immunological, post-infective, genetic, or aspiration-related drivers. These distinct etiologies demand tailored diagnostic hierarchies and divergent therapeutic interventions.

The global burden of non-cystic fibrosis bronchiectasis continues to climb across aging healthcare systems. This epidemiological rise stems from population aging, widespread adoption of high-resolution chest computed tomography (CT), improved survival rates following severe pulmonary infections, and heightened recognition of secondary bronchiectasis in systemic inflammatory and immune-mediated disorders (3). Registries and large population-based cohorts in China emphasize the expanding public health relevance of this syndrome, highlighting substantial regional variations in both etiology and microbial colonization (4). Consequently, traditional diagnostic touchstones no longer suffice. Traditional, purely descriptive radiological reporting no longer fulfills the requirements of targeted clinical care.

Current international consensus guidelines dictate confirmation via high-resolution CT (HRCT), objective severity staging, meticulous exacerbation tracking, screening for modifiable underlying causes, and serial microbiological surveillance (5–8). These steps form a baseline standard. Yet, conventional clinical frameworks frequently overlook critical pathological variations between patients. Visual CT scoring remains notoriously subjective. Sputum cultures often return negative results despite active, tissue-destructive microbial processes. Similarly, systemic blood tests rarely mirror the localized microenvironment of airway inflammation. Standard clinic visits offer nothing more than intermittent, disconnected snapshots of a disease that fluctuates continuously over time. These systemic shortcomings expose the pressing need for objective, precision diagnostic metrics.

Therefore, the primary objective of this review is to critically evaluate three core diagnostic domains—advanced imaging, microbiome-driven microbiology, and systemic inflammatory biomarkers—within the context of evolving bronchiectasis management. By synthesizing recent evidence, we identify actionable diagnostic strategies, highlight persistent knowledge gaps, and outline a framework for translating precision diagnostics into equitable clinical practice across diverse healthcare settings. Our analysis shifts focus entirely toward this pragmatic clinical utility.

## 2 Scope of the review

This article provides a critical narrative review of contemporary diagnostic and evaluative tools for adult non-cystic fibrosis bronchiectasis. Our target audience includes respiratory clinicians, translational scientists, radiologists, microbiologists, and clinical researchers. We prioritized peer-reviewed cohort data, international guidelines, consensus statements, randomized controlled trials, and official regulatory filings published up to May 2026. This text does not function as a systematic review or meta-analysis. We calculated no pooled effect estimates. Instead, we synthesize heterogeneous evidence with a strict focus on biological plausibility, reproducibility, and real-world deployment readiness.

Our critical lens is deliberately conservative. Several widely discussed technologies—specifically radiomics, artificial intelligence (AI)-driven image analysis, metagenomic sequencing, and wearable-based exacerbation forecasting—are currently transitioning from isolated research environments into active clinical workflows. Evidence here remains preliminary. Where data rely on single-center, retrospective, or unvalidated cohorts, we state these limitations explicitly. We discarded all quantitative assertions lacking verifiable primary sources.

A systematic literature search of PubMed and Web of Science (2020–2026) was conducted using the following terms: “bronchiectasis,” “precision medicine,” “biomarker,” “microbiome,” and “imaging.” Studies were included if they addressed diagnostic innovations, biomarker validation, or clinical classification in adult bronchiectasis populations. Priority was given to randomized controlled trials, large cohort studies, meta-analyses, and multinational consensus statements. Ongoing clinical trials were identified through ClinicalTrials.gov.

## 3 Imaging assessment beyond descriptive HRCT

HRCT remains the diagnostic baseline for this condition. The established radiological signature comprises an elevated broncho-arterial ratio, impaired bronchial tapering, peripheral airway visibility near the costal pleura, bronchial wall thickening, mucus impaction, mosaic perfusion, tree-in-bud opacities, and segmental volume loss (5, 7). Classical scoring systems, notably the Bhalla index and the subsequent Bronchiectasis Radiologically Indexed CT Score (BRICS), provided foundational frameworks to standardize radiological descriptions and enable research stratifications (9, 10). Manual scoring remains inherently cumbersome. These tools demand significant reader expertise and suffer from substantial inter-observer variability, particularly when evaluating mild cylindrical changes or subtle longitudinal progression.

Practical clinical optimization begins with disciplined standardization rather than complex novel algorithms. Proper CT acquisition requires strict documentation of slice thickness, inspiratory effort, reconstruction kernels, radiation metrics, and expiratory trapping sequences. Radiologists must systematically document spatial distribution, wall thickness, mucus load, atelectasis, comorbid emphysema, air trapping, specific nodules, and features indicating distinct etiologies. These etiologies include allergic bronchopulmonary aspergillosis ((ABPA)), nontuberculous mycobacterial (NTM) disease, post-tuberculous architectural destruction, primary ciliary dyskinesia (PCD), or chronic aspiration. Longitudinal evaluations must carefully differentiate true structural progression from technical variations in inspiratory volume or scanner physics.

Artificial intelligence-assisted quantitative CT (AI-QCT) attempts to mitigate human error by automating airway segmentation and measuring broncho-arterial metrics across thousands of branches simultaneously. Data from the COPDGene study demonstrate how automated CT analysis can successfully quantify bronchiectasis-like airway alterations and map their correlation with objective respiratory outcomes (11). Such digital toolsets could theoretically standardize longitudinal surveillance, eliminate reporting bias, and flag early, spatially isolated disease that eludes human visual inspection.

Significant technical caveats remain unresolved. AI-derived airway metrics vary wildly based on CT protocols, inspiratory effort, airway generation depth, vessel segmentation logic, reconstruction kernels, and the specific demographic distributions of the training datasets. A broncho-arterial ratio threshold optimized for one proprietary scanner or ethnic cohort frequently fails when applied to alternative hardware or distinct disease contexts. Consequently, we argue that AI-QCT must be utilized strictly as an adjunct to expert chest radiology rather than a standalone diagnostic standard. Routine clinical adoption remains challenging without extensive multicenter validation, transparent algorithmic benchmarking, cross-scanner calibration, and hard, clinically meaningful outcome endpoints.

Radiomics extends traditional image analysis by extracting thousands of high-dimensional mathematical features from conventional CT scans, analyzing texture, intensity distributions, shape descriptors, and spatial heterogeneity. In theory, unique radiomic signatures can map hidden patterns linked to regional mucus burden, localized air trapping, persistent infection, or underlying inflammatory activity. Methodological discipline here is critical. Stable feature extraction requires highly standardized image acquisition, precise segmentation boundaries, feature harmonization across platforms, and external validation (12). Radiomic applications in bronchiectasis remain immature compared to direct quantitative airway anatomy. We recommend extreme caution when interpreting claims of high predictive accuracy unless they are backed by independent replication cohorts and fully transparent model reporting.

Functional imaging offers a valuable adjunct when clinical symptoms, physiological lung function, and structural anatomy conflict. Hyperpolarized gas magnetic resonance imaging (MRI), utilizing helium-3 or xenon-129, uncovers regional ventilation defects and subtle small-airway dysfunction. Comparative investigations in chronic obstructive lung diseases reveal that free-breathing proton MRI and hyperpolarized gas MRI provide localized functional data completely missed by standard spirometry (13). These modalities will not replace HRCT. Instead, their utility lies in translational research, early-stage pathology detection, drug-response tracking, and monitoring patients where cumulative radiation exposure presents a legitimate clinical concern.

## 4 Biomarkers and inflammatory endotyping

Bronchiectasis has historically been categorized as a neutrophil-dominated pathology. This paradigm holds true for the vast majority of patients suffering from chronic bacterial colonization, persistent mucus retention, and frequent clinical exacerbations. Active sputum neutrophil elastase represents one of the most thoroughly validated and clinically informative airway biomarkers investigated to date. The available data are noteworthy. Large-scale observational data confirm that elevated neutrophil elastase activity closely correlates with heightened exacerbation risks and accelerated lung-function decline (14). This supports its role as an indicator of localized disease activity and a viable pharmacodynamic biomarker for emerging neutrophil-targeted therapeutics.

Despite this biological relevance, routine clinical deployment of sputum neutrophil elastase remains virtually non-existent. Formidable practical barriers persist. These include variations in sputum induction, prolonged processing times, unpredictable sample dilution, conflicting assay platforms, storage instabilities, and poorly defined diagnostic thresholds. Biomarker standardization is non-negotiable. A laboratory metric that cannot be reliably replicated across institutions holds no real value for clinical decision-making. Future prospective trials must thoroughly document pre-analytical handling protocols, precise patient exacerbation status, concurrent antibiotic exposure, background microbiological status, and whether the biomarker score actively alters therapeutic selection or predicts clinical response.

Eosinophilic bronchiectasis represents a major challenge to the historical assumption that all bronchiectasis inflammation is purely neutrophilic. A granular European multicohort study successfully isolated an eosinophilic subgroup, demonstrating that peripheral blood eosinophil counts can identify patients harboring a distinct, type 2-driven inflammatory profile (15). Therapeutic implications are immediate. While international guidelines strongly discourage the indiscriminate use of inhaled corticosteroids in generic bronchiectasis, these agents become highly rational choices when managing patients presenting with coexisting asthma, allergic bronchopulmonary aspergillosis, eosinophilic chronic obstructive pulmonary disease (COPD), or a highly reproducible type 2 inflammatory signature.

Peripheral blood eosinophil metrics are highly attractive due to their low cost and universal availability. However, clinical interpretation demands extreme caution. Eosinophil counts fluctuate dynamically in response to active bacterial infections, systemic corticosteroid administration, biological therapies, smoking habits, occult parasitic infections, and concurrent asthmatic flares. We warn against overinterpreting an isolated, single-point blood eosinophil value. A superior approach integrates longitudinal eosinophil dynamics with clinical symptom patterns, historical exacerbation rates, serial spirometry, fractional exhaled nitric oxide (FeNO), detailed allergy profiles, and high-resolution imaging features. The core clinical question is straightforward. Do eosinophils expose a modifiable trait that changes the treatment pathway? (16).

Systemic inflammatory markers—including C-reactive protein, total leukocyte counts, the neutrophil-to-lymphocyte ratio, serum albumin, and multi-component frailty or nutritional indexes—help contextualize baseline physiological risk, particularly within aging or multimorbid patient populations. Contextual risk tracking is essential. These markers operate best as components of an integrated risk matrix rather than disease-specific diagnostic readouts. Consequently, our analysis supports a layered, pragmatic biomarker strategy for modern bronchiectasis management. Use inexpensive systemic markers to establish baseline vulnerability. Deploy validated airway biomarkers to track localized disease activity when feasible. Reserve resource-intensive, targeted inflammatory endotyping exclusively for clinical scenarios where the selection of a specific therapy hinges directly on the molecular result.

This diagnostic duality underscores an important conceptual distinction (17): assays that perform well in controlled research environments—where sample handling, patient selection, and outcome ascertainment are standardized—may not translate seamlessly into real-world clinical workflows. Clinicians must therefore evaluate biomarker evidence not only by statistical significance but also by pre-analytical robustness, assay reproducibility, and incremental value beyond routine clinical parameters. Future studies should prioritize pragmatic trial designs that embed biomarker assessment within routine care pathways.

## 5 Molecular microbiology and the lung microbiome

Microbiota dynamics drive bronchiectasis pathology. Chronic airway infection directly dictates clinical symptom severity, annual exacerbation frequencies, localized tissue inflammation, overall quality of life, and long-term mortality tracking. While standard sputum culture persists as our frontline surveillance mechanism due to low costs, universal accessibility, and its unique capacity to isolate viable vegetative organisms for phenotypic antimicrobial susceptibility testing, its diagnostic utility remains constrained by inherent methodology limitations. Specifically, diagnostic sensitivity decreases significantly following empirical antibiotic exposure, which frequently allows fastidious or obligate anaerobic species to evade standard detection. Furthermore, prolonged turnaround times often delay timely therapeutic adjustments, meaning that a culture-negative status may inadvertently mask an active, ongoing infection. Most importantly, an isolated streak plate cannot map the architectural complexity of the collective airway microbial ecosystem. Clinicians must not conflate an empty petri dish with a sterile, quiescent airway environment.

High-throughput microbiome sequencing has substantially revised historical paradigms, reframing bronchiectasis from a state of episodic pathogen invasion to one of perpetual ecological dysbiosis. Early genomic profiling demonstrated that even during periods of apparent clinical stability, the bronchiectatic airway maintains a complex, highly dynamic polymicrobial ecosystem that undergoes profound compositional and quantitative shifts during acute exacerbations (18). Recent data confirm that longitudinal microbiota stratification accurately forecasts upcoming exacerbation risks and maps directly onto long-term patient survival curves (19, 20). These microbial ecosystems undergo profound compositional and quantitative shifts during acute exacerbations, particularly characterized by *Pseudomonas aeruginosa* dominance that triggers unchecked neutrophilic recruitment, accelerated structural architectural destruction, and a precipitous decline in independent clinical outcomes (21).

Molecular diagnostic toolsets span a spectrum. Targeted multiplex polymerase chain reaction (PCR) arrays and localized next-generation sequencing assays confine their search to predetermined pathogen panels or specific antimicrobial resistance alleles. This narrow scope permits rapid, high-yield diagnostic returns during acute clinical deterioration. Metagenomic next-generation sequencing (mNGS) casts a far wider net. By indiscriminately extracting and sequencing all total genomic DNA and RNA from an unselected clinical sample, this open-ended platform maps millions of raw sequencing reads against vast, continuously updated microbial reference databases. Enthusiasm has, in some areas, outpaced the available evidence. While the broader utility of clinical mNGS for undifferentiated pathogen detection is well documented (22), emerging bronchiectasis-specific data suggest its practical deployment must be tightly restricted. Our analysis shows that mNGS should be reserved for scenarios where conventional cultures fail, occult or atypical pathogens are strongly suspected, or when analyzing invasive bronchoalveolar lavage fluid from severe, treatment-refractory cohorts (23, 24). We observe similar limitations regarding genotypic resistance screening. Detecting a resistance gene accelerates preliminary clinical decisions, but it cannot predict true phenotypic expression or establish absolute minimum inhibitory concentrations (25). The underlying biology is often more nuanced.

The primary interpretative hurdle for metagenomic next-generation sequencing (mNGS) shifts from mere analytical detection to true clinical attribution. Given that the respiratory tract is never sterile, diseased bronchiectatic airways harbor a complex, structural flux of background colonizers, harmless commensals, residual nucleic acids from dead bacteria following recent antibiotic exposure, and exogenous environmental contaminants introduced during specimen sampling or laboratory processing. Context dictates clinical meaning. Consequently, an actionable mNGS readout requires meticulous documentation of sample types, stringently defined quality metrics, total microbial read counts alongside relative abundance data, negative background controls, biological plausibility of the identified genus, and prospective genotypic resistance determinants. We must mandate clear statements of inherent interpretative limitations. Clinicians must integrate these molecular data with clinical symptomatology, pyrexia, sputum purulence, systemic inflammatory markers, high-resolution imaging, historical microbiology, and proximal antibiotic exposure. Sequencing cannot dictate therapy automatically. Knee-jerk resorting to broad-spectrum antimicrobials based on raw molecular readouts alone threatens global antimicrobial stewardship.

High-throughput microbiome characterization actively dismantles the historical, reductionist single-pathogen paradigm. Given the vast pathological heterogeneity, one individual presenting with chronic, recurrent *Pseudomonas aeruginosa* isolation often harbors a highly stable, low-diversity, perpetually inflammation-prone microbial ecosystem, whereas another clinically identical patient may feature rich anaerobic syndromic communities, viral triggers, or volatile, intermittent *Haemophilus influenzae* dominance. Future diagnostic platforms will likely merge conventional cultivation techniques, focused multiplex molecular assays, untargeted mNGS, and mathematical microbiome alpha- and beta-diversity indices (Table 1). This integration remains unstandardized. Transitioning these multi-omic data to the bedside demands strict collection harmonization and robust, prospective evidence demonstrating that microbiome-guided therapeutic alterations yield superior long-term clinical outcomes.

**Table 1:** Comparison of microbiological diagnostic tools in bronchiectasis.

Feature	Conventional sputum culture	Targeted molecular testing (multiplex PCR/tNGS)	Metagenomic NGS (mNGS)
Analytical scope	Cultivable bacteria/fungi; strongest for common organisms	Predefined pathogen panel ± resistance genes	Unbiased detection of bacterial, fungal, viral, and atypical nucleic acids
Sensitivity/yield	Reduced post-antibiotics/poor sputum; misses fastidious/anaerobic organisms	Higher yield for included targets; panel-dependent	High analytical sensitivity, especially in BALF; clinical specificity depends on contamination control and interpretation (22–24)
Time to actionable result	48–96 h (longer for slow-growing organisms)	Hours to 1–2 days	2–4 days (rapid pipelines developing)
Resistance information	Phenotypic susceptibility testing available and interpretable	Rapid genotypic detection of selected determinants	Broad genotypic resistance inference is possible, but clinical interpretation requires organism abundance and gene-context assessment (25)
Effect of prior antibiotics	Yield substantially reduced (non-viable organisms do not grow)	Less affected (nucleic acid persists)	Less affected analytically; DNA/RNA from non-viable organisms complicates interpretation
Strengths and indications	Low cost, routine availability; baseline surveillance and chronic infection management	Fast, focused; early pathogen-directed decisions during exacerbations	Detection in culture-negative/severe/atypical disease; specialist evaluation of difficult cases
Limitations	Slow turnaround, variable sensitivity, sputum quality concerns, limited ecological data	Panel-dependent blind spots; cannot fully characterize microbiome	Cost, contamination, colonization-vs.-infection uncertainty, database bias, and limited standardization of reporting thresholds

BALF, bronchoalveolar lavage fluid; DNA, deoxyribonucleic acid; mNGS, metagenomic next-generation sequencing; PCR, polymerase chain reaction; RNA, ribonucleic acid; tNGS, targeted next-generation sequencing.

## 6 Etiological investigation and deep phenotyping

Diagnostic investigations in bronchiectasis achieve clinical utility only when they expose underlying etiologies that directly modify therapeutic management. International consensus guidelines mandate systematic screening for humoral immune deficiencies, allergic bronchopulmonary aspergillosis (ABPA), nontuberculous mycobacterial (NTM) disease, concomitant asthma, chronic obstructive pulmonary disease (COPD), systemic autoimmune conditions, inflammatory bowel disease, occult chronic aspiration, primary ciliary dyskinesia (PCD), and *CFTR*-related dysfunctions (5–7). Causes dictate outcomes. Data from the US Bronchiectasis Research Registry emphasize the extreme phenotypic diversity characterizing adult cohorts, confirming that comorbid obstructive airway pathologies and chronic microbial colonization represent the clinical rule rather than the exception (26).

Deep phenotyping must be clinically directed, as unfocused screening strategies may impose unnecessary financial and logistical burdens on healthcare systems. The co-occurrence of early-onset diffuse disease, neonatal respiratory distress, recalcitrant otitis media, chronic rhinosinusitis, unexplained subfertility, situs inversus, or an established family history must trigger immediate, targeted screening for underlying primary ciliary dyskinesia. Conversely, histories marked by recurrent pyogenic infections, opportunistic or unusual pathogens, blunted vaccine challenges, or depressed baseline serum immunoglobulin concentrations require prompt evaluation for primary humoral immune deficiencies. Spatial distribution guides suspicion. Upper-lobe dominant disease featuring extensive mucus impaction and refractory asthmatic symptoms strongly points toward allergic bronchopulmonary aspergillosis (27). Isolated, localized architectural damage, by contrast, suggests mechanical airway obstruction, remote foreign body aspiration, focal post-infective scarring, or specific congenital anatomical abnormalities.

Widespread commercial accessibility has democratized clinical genetic testing. Yield remains low without high pre-test probability. While expansive high-throughput sequencing panels readily identify clear pathogenic variants within *CFTR* or ciliary assembly genes, the frequent discovery of variants of uncertain significance (VUS) drives profound clinical ambiguity. Context resolves genetic doubt. Genetic data are uninterpretable unless aligned with granular clinical phenotypes, sweat chloride quantitative assessments, nasal nitric oxide metrics, high-speed video ciliary beating analyses, systemic immunological baselines, and extended family pedigrees. Genomics is a double-edged sword. Precision medicine discourages the indiscriminate generation of raw genetic datasets. It demands the strategic selection of diagnostic assays possessing clear, biologically plausible pathways to target-specific clinical management.

## 7 Treatable traits, digital health, and therapeutic consequences

The treatable-traits paradigm bridges the gap between multi-omic diagnostic complexity and practical, bedside medical decision-making. Rather than treating bronchiectasis as an undifferentiated, monolithic diagnostic label, clinicians must systematically isolate specific, overlapping pulmonary, extrapulmonary, and behavioral components that remain uniquely amenable to targeted modification (28, 29). Pulmonary traits encompass chronic *Pseudomonas* colonization, accelerated exacerbation phenotypes, severe mucus hypersecretion, fixed or reversible airflow limitation, localized nontuberculous mycobacterial disease, or polarized neutrophilic or eosinophilic inflammatory endotypes. Extrapulmonary axes introduce further clinical complexity. These traits require active screening for gastroesophageal reflux with occult aspiration, chronic rhinosinusitis, systemic immunodeficiencies, physical frailty, clinical anxiety or depression, severe malnutrition, and deep sedentary behavior. Finally, behavioral dimensions demand close interrogation. We frequently observe that poor adherence to airway clearance regimens, critical inhaler handling errors, active tobacco dependence, and socioeconomic barriers to specialist access undermine otherwise optimal therapeutic strategies.

Mechanical airway clearance forms the bedrock of long-term therapy. Systematic Cochrane evaluations confirm that structured airway clearance techniques significantly alleviate respiratory symptoms and improve health-related quality of life indices in non-cystic fibrosis cohorts, despite significant trial heterogeneity and a persistent scarcity of long-term survival data (30, 31). This operational reality reshapes routine diagnostic follow-ups. Clinicians cannot simply assume compliance or efficacy. Sputum volume, localized mucus impaction, cough mechanics, and physical patient adherence must be dynamically re-evaluated at every clinical encounter. Objective radiological evidence of progressive mucus plugging or subsegmental atelectasis provides a clear mandate to escalate mechanical clearance regimens, initiate hypertonic saline nebulization, request specialized chest physiotherapy review, or intensify targeted antimicrobial interventions.

Physical deconditioning represents a similarly impactful modifiable trait. Formal pulmonary rehabilitation significantly enhances functional exercise capacity and mitigates baseline dyspnea in non-cystic fibrosis cohorts, though sustaining these clinical gains depends entirely on continuous physical activity and structured self-management frameworks (32). Systematic diagnostic profiling must extend beyond traditional microbiological assays and anatomic thoracic imaging. Our analysis shows that systematic deployment of the 6-min walk distance test, validated dyspnea scales, objective fatigue assessments, frailty indexes, nutritional metrics, and comorbidity profiling isolates a distinct subgroup of patients whose functional decline is driven not by active infectious processes, but by severely depleted physiological reserve.

**Figure 1:**
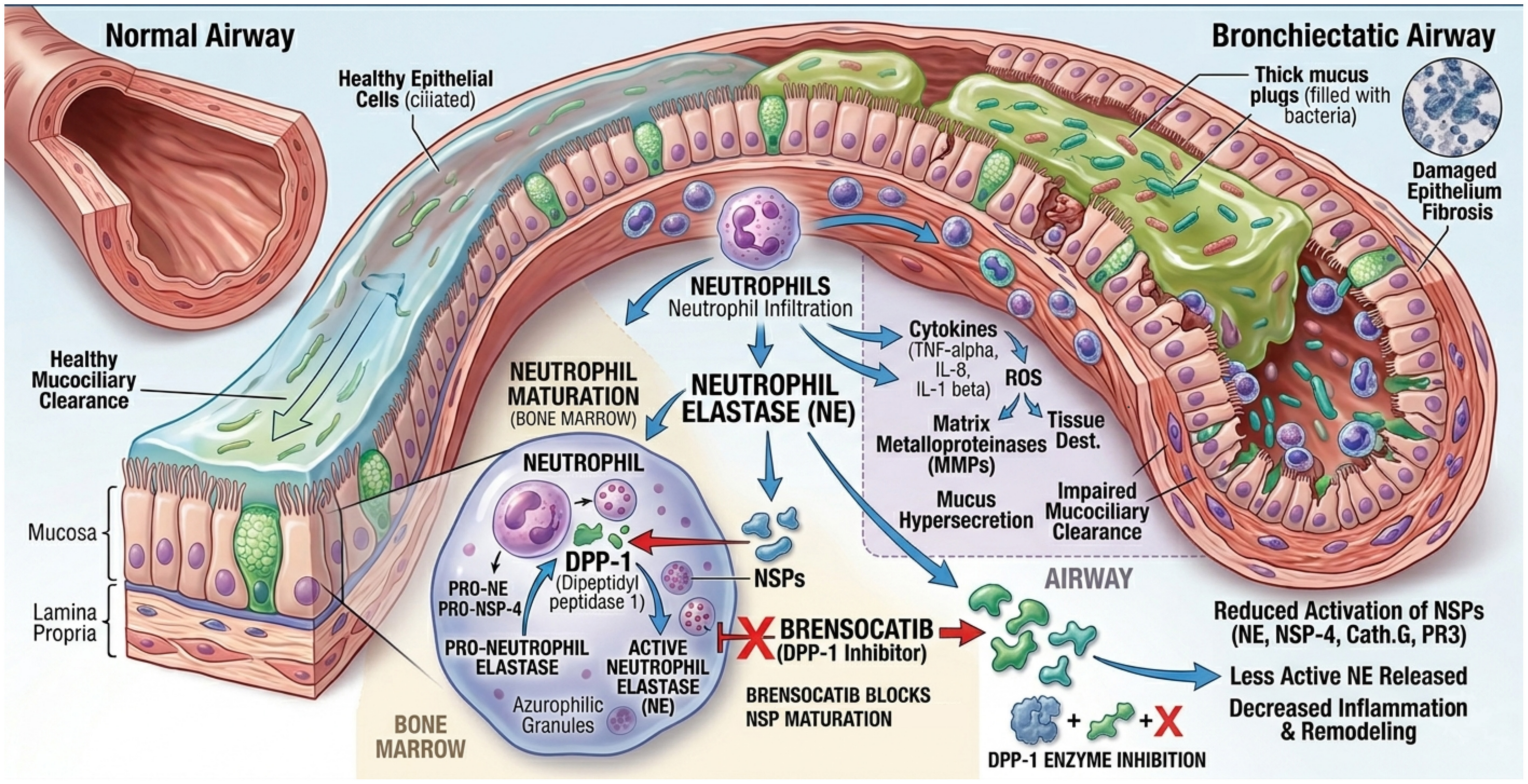
Pathophysiology of neutrophil-driven inflammation in bronchiectasis and the mechanism of action of brensocatib (DPP-1 inhibitor). The schematic illustrates the stark contrast between a normal airway **(left)** with healthy mucociliary clearance and a bronchiectatic airway **(right)** characterized by neutrophil infiltration, chronic bacterial colonization, and tissue remodeling. Inset: within the bone marrow during neutrophil maturation, Dipeptidyl peptidase 1 (DPP-1) cleaves and activates pro-neutrophil elastase into active neutrophil elastase (NE) stored in azurophilic granules. Brensocatib selectively inhibits DPP-1 activity, blocking the maturation of these neutrophil serine proteases (NSPs). Consequently, neutrophils released into the airway secrete less active NE, effectively decoupling the pathogenic vicious vortex of inflammation, mucus hypersecretion, and structural airway damage.

The most notable recent validation of endotype-directed pharmacotherapy comes from dipeptidyl peptidase-1 (DPP-1) inhibition. Targeting endotypes works. In the phase 2 WILLOW trial, the novel oral DPP-1 inhibitor brensocatib successfully suppressed neutrophil serine protease activity in sputum, significantly extending the time to first clinical exacerbation (33). The subsequent multicenter phase 3 ASPEN trial confirmed these findings, demonstrating that once-daily brensocatib administration resulted in a significantly lower annualized rate of pulmonary exacerbations compared with placebo across a broad bronchiectasis population (34). Regulatory frameworks have responded rapidly (Figure 1). In the United States, brensocatib has received regulatory labeling approval for managing non-cystic fibrosis bronchiectasis in adults and pediatric patients aged 12 years and older (35). This pharmacological breakthrough provides a strong clinical rationale for the routine quantification and deep characterization of neutrophilic airway inflammation. Questions persist. Moving forward, the respiratory community must resolve uncertainties regarding optimal patient sub-selection, long-term safety profiles, absolute cost-effectiveness, and real-world implementation alongside baseline mechanical clearance and cycling antibiotic regimens (36).

Digital health technologies promise to enhance the temporal resolution of longitudinal disease surveillance. Continuous passive monitoring captures hidden trends. Smartphone-enabled symptom diaries, home-based micro-spirometry, automated acoustic cough-frequency monitors, serial pulse oximetry, wearable physical activity trackers, and synchronous telemedicine consultations collectively harvest granular physiological shifts occurring entirely outside traditional clinic appointments. Digital remote monitoring networks operate as high-utility adjuncts in advanced bronchiectasis care, especially when deployed to protect high-exacerbation phenotypes or geographically isolated individuals lacking direct access to tertiary specialty centers (37). A measured degree of caution remains appropriate. Digital surrogate endpoints must undergo rigorous prospective validation against hard, clinically meaningful outcomes before widespread clinical integration. A summary of advanced diagnostic dimensions is presented in Table 2. Data volume is not utility. We observe an urgent need for multi-center data defining which digital signals reliably forecast imminent clinical deterioration, how algorithmic false alarms can be safely triaged, and how remote care frameworks can be seamlessly delivered without inflating clinical workloads or systematically marginalizing vulnerable patients suffering from low digital literacy.

**Table 2:** Comparison of advanced diagnostic dimensions.

Domain	Representative tool	Evidence base	Clinical value	Current limitation
Imaging	AI-assisted quantitative CT	Validated against large cohorts for airway quantification and outcome prediction (11)	Objective airway mapping, longitudinal comparison, reduced reader variability	Scanner variation, threshold uncertainty, limited external validation
Microbiology	Targeted molecular testing and mNGS	Value in culture-negative/severe/refractory cases (23, 24)	Earlier pathogen detection, broader coverage, resistance-gene information	Colonization vs. infection ambiguity, cost, reporting heterogeneity
Inflammatory endotype	Sputum neutrophil elastase, blood eosinophils	Neutrophil elastase predicts exacerbations and lung-function decline; eosinophilic bronchiectasis is a reproducible subgroup (14, 15)	Risk stratification and endotype-directed therapy selection	Assay standardization, threshold variability, incomplete therapeutic algorithms
Etiology	Guideline-guided immunology, ABPA, NTM, CFTR/PCD, aspiration and autoimmune evaluation	Guideline-supported systematic etiological assessment (5–7)	Cause-specific treatment, family counseling, infection prevention	Variable access, specialist interpretation, and uncertain yield of broad genetic panels in unselected adults
Digital monitoring	Telemedicine, symptom diaries, home physiology and cough/activity tracking	Feasible for follow-up in selected patients (37)	Earlier exacerbation detection, adherence support, specialist access	Validated endpoints, privacy safeguards, workflow integration, equity concerns

ABPA, allergic bronchopulmonary aspergillosis; AI, artificial intelligence; CFTR, cystic fibrosis transmembrane conductance regulator; CT, computed tomography; mNGS, metagenomic next-generation sequencing; NTM, nontuberculous mycobacteria; PCD, primary ciliary dyskinesia.

The divergence between promising preclinical data and negative trial results warrants reflection. The ORBIT-3 and ORBIT-4 trials of inhaled ciprofloxacin failed to meet their primary endpoints despite strong *in vitro* activity, highlighting that microbiological eradication does not guarantee clinical benefit (38–40). These failures collectively suggest that single-pathogen targeting may be insufficient in a disease characterized by polymicrobial colonization and host heterogeneity. Future therapeutic trials should incorporate biomarker-based patient stratification—for example, selecting patients with elevated neutrophilic activity for anti-neutrophil interventions—rather than enrolling unselected bronchiectasis populations.

## 8 Multimodal integration, clinical translation, and global implementation

Multimodal data integration dictates the real-world efficacy of advanced diagnostics. Isolated data points fail. When quantitative high-resolution computed tomography (HRCT) captures macrostructural deterioration, molecular microbiological platforms characterize localized pathogens, airway biomarkers expose immediate inflammatory kinetics, etiological screenings unearth baseline disease drivers, and digital health networks log longitudinal real-time fluctuations, they must act in concert. Fractional analysis breeds error. Our analysis shows that orchestrating these separate streams shifts the clinical focus from static anatomical description to a dynamic, multi-dimensional trajectory, exposing the most actionable modifiable trait for any given patient.

We advocate a staged, rather than indiscriminate, diagnostic hierarchy. Basic screening suffices initially. For patients presenting with mild, stable, culture-positive disease, standard HRCT, baseline spirometry, meticulous exacerbation histories, and routine guideline-directed etiological tracking provide sufficient clinical clarity without escalating to high-throughput sequencing or radiomics. Conversely, complex cohorts—marked by frequent unexplained exacerbations, culture-negative clinical deterioration, atypical structural patterns, or repeated empirical antibiotic failures—demand aggressive diagnostic escalation via bronchoscopy-directed sampling, metagenomic sequencing, and expert multi-disciplinary review. Phenotypes dictate the therapeutic pathway. Intense sputum burden paired with extensive mucus impaction demands mechanical airway clearance prioritization over alternative antimicrobials, whereas highly reproducible eosinophilic profiles necessitate direct secondary evaluation for coexisting asthmatic endotypes or allergic bronchopulmonary aspergillosis.

Regional heterogeneity challenges Western-centric diagnostic paradigms. Geographic and environmental factors influence clinical presentation (41). In China, the clinical presentation of bronchiectasis diverges substantially from North American and European cohorts due to a high prevalence of post-tuberculous architectural destruction, distinct environmental particulate exposures, variable healthcare access, and unique regional pathogen distributions. The BE-China registry (42, 43) and similar regional initiatives, such as the Indian registry (44, 45), address this knowledge gap by generating prospective, multicenter data that map the regional atlas of patient characteristics, overlapping comorbidities, and localized microbiology. We observe that diagnostic models derived exclusively from Western cohorts regularly fail to generalize across large Asian populations, making indigenous registry data indispensable for defining localized phenotypes.

International collaborative platforms such as the European Multicentre Bronchiectasis Audit and Research Collaboration (EMBARC) (31, 46, 47) and the newly established Child-BEAR-Net (Bronchiectasis in Children and Adolescents Research Network) have demonstrated the power of standardized data collection across diverse populations. These registries have enabled risk-stratification model validation, treatment pattern analysis, and identification of regional etiological differences that would be impossible to discern from single-center studies. Expanding such networks to include low- and middle-income country (LMIC) representation remains a priority for global bronchiectasis research.

Implementing these models requires a tiered infrastructure. Resource allocation must match clinical capacity. Primary and secondary care frameworks should prioritize early syndrome recognition, standardized HRCT confirmation, basic sputum cultivation, vaccination updates, and clear triggers for specialist referral. Tertiary academic centers can then reserve their resources for deep phenotyping, invasive bronchoscopy, complex immunologic panels, mycobacterial isolation, genetic counseling, and untargeted sequencing assays. Bridging these distinct healthcare tiers via national registries and digital telemedicine infrastructure ensures continuous longitudinal monitoring, actively narrowing the care equity gap between affluent urban centers and resource-limited regional clinics.

However, translating these tiered diagnostic models into global clinical practice requires addressing stark resource disparities. The advanced diagnostic innovations discussed above—such as clinical proteomics, metagenomic sequencing, and computational imaging—remain largely confined to specialized academic centers in high-income countries (HICs). For the majority of bronchiectasis patients worldwide who receive care in LMICs, even basic spirometry and sputum culture may be unavailable. Without deliberate implementation strategies, the precision medicine revolution risks widening, rather than narrowing, global health disparities.

Pragmatic adaptation is possible. Simplified diagnostic algorithms using widely available parameters (blood eosinophil counts, basic spirometry, chest X-ray) can risk-stratify patients when advanced tools are inaccessible. Partnerships with national tuberculosis programs—which already maintain microscopy and culture infrastructure in many LMICs—offer a platform for bronchiectasis diagnostic capacity building. Essential medicines lists should include affordable inhaled therapies and mucoactive agents alongside novel biologics. Most urgently, prospective epidemiological studies in LMIC populations are needed to establish region-specific prevalence estimates, etiological profiles, and outcome determinants, since data derived predominantly from European and North American cohorts may not generalize.

## 9 Future directions and implementation barriers

Definitive clinical translation faces substantial implementation hurdles. Technical harmonization represents the primary obstacle. Imaging metrics require cross-platform standardizations governing image acquisition protocols, automated segmentation logics, airway generation boundaries, and clinical reporting templates. Similarly, molecular microbiology platforms lack universal consensus regarding quantitative thresholds for clinical significance, strict contamination filters, and actionable genotypic resistance reporting. Airway biomarkers suffer from unharmonized pre-analytical sputum processing, while digital health frameworks remain decoupled from validated clinical intervention algorithms.

Moving beyond technical uniformity, external validation should be required. Predictive models frequently suffer from extreme overfitting within single-center or demographically narrow training datasets. Algorithms optimized for a single proprietary scanner configuration or a localized microbial ecosystem regularly collapse when deployed externally. We argue that future prospective trials must rigorously report algorithmic calibration, spatial discrimination, decision-curve analyses, and distinct subgroup performance stratified by age, sex, biological ethnicity, tobacco exposure, comorbid obstructive airway pathologies, post-tuberculous anatomy, and colonization profiles. Validation within underrepresented Asian cohorts represents a fundamental scientific necessity rather than an optional geographic extension.

True clinical utility demands pragmatic evaluation. Technical novelty cures nothing. A diagnostic platform may achieve remarkable mathematical precision yet remain a functional failure if its outputs fail to modify therapeutic decisions or alter patient-centric outcomes. We need multicenter, randomized implementation trials to determine whether AI-guided monitoring, mNGS-driven antimicrobial selections, biomarker-stratified anti-inflammatory interventions, or wearable-based early warning networks actively minimize annualized exacerbation rates, reduce cumulative antibiotic exposure, prevent hospitalizations, attenuate lung-function decline, or lower systemic healthcare expenditures. Cost-effectiveness must be benchmarked directly against diagnostic accuracy.

Administrative governance and provider literacy cap these logistical barriers. High-dimensional datasets generated by genomic sequencing, multi-omic microbiome profiling, and continuous wearable networks introduce complex data privacy, ownership, and cross-border sharing vulnerabilities. Provider literacy introduces an equally steep hurdle; frontline clinicians often lack the specialized training required to interpret complex algorithmic probability outputs, raw metagenomic sequencing readouts, or shifting biomarker panels. Black boxes invite clinical error. An opaque, uninterpretable computational report that cannot be translated into a transparent patient communication strategy or an actionable bedside intervention holds zero clinical value. Precision medicine in this field will succeed only when advanced diagnostic tools remain universally interpretable, promote equity, and link directly to interventions that matter to the patient.

## 10 Conclusions

Bronchiectasis evaluation is undergoing an irreversible shift away from purely descriptive, anatomy-based models toward a dynamic precision paradigm that characterizes structural destruction, microbial ecology, localized inflammation, host etiology, comorbidities, and patient behavior as interacting, modifiable axes. Descriptive medicine alone is no longer sufficient. High-resolution CT remains the diagnostic anchor, but automated quantitative imaging and algorithmic airway tracking improve longitudinal reproducibility. Conventional cultivation preserves its role in phenotypic resistance testing, yet molecular sequencing and microbiome metrics expose hidden ecological dysbiosis that routine culture plates miss entirely. Similarly, sputum neutrophil elastase and peripheral blood eosinophil counts successfully isolate distinct inflammatory endotypes, a capability that gains clinical importance as targeted, endotype-directed pharmacotherapies emerge. Wearable sensors and digital inputs may extend clinical surveillance into the home, provided their signals undergo strict validation and integrate smoothly into practical care algorithms.

The immediate priority demands disciplined translation. Novel diagnostic modalities must be rigorously standardized, externally replicated, regionally calibrated, and explicitly tested for real-world clinical utility. Within vast, heterogeneous healthcare networks like China, multi-center registries, tiered medical delivery systems, and localized validation frameworks represent key pathways toward equitable implementation. Ultimately, precision medicine for bronchiectasis must not be evaluated by the sheer volume of high-dimensional data collected. Success will likely depend on whether these advanced diagnostic tools enable earlier clinical recognition, optimize therapeutic selection, minimize exacerbations, halt structural disease progression, and preserve long-term patient quality of life.

## References

[1] Chalmers JD Chang AB Chotirmall SH Dhar R McShane PJ. Bronchiectasis. Nat Rev Dis Primers. (2018) 4:45. doi: 10.1038/s41572-018-0042-3 30442957

[2] Cole PJ. Inflammation: a two-edged sword—the model of bronchiectasis. Eur J Respir Dis Suppl. (1986) 147:6–15. 3533593

[3] McShane PJ Naureckas ET Tino G Strek ME. Non-cystic fibrosis bronchiectasis. Am J Respir Crit Care Med. (2013) 188:647–56. doi: 10.1164/rccm.201303-0411CI 23898922

[4] Feng J Sun L Sun X Xu L Liu L Liu G . Increasing prevalence and burden of bronchiectasis in urban Chinese adults, 2013–2017: a nationwide population-based cohort study. Respir Res. (2022) 23:111. doi: 10.1186/s12931-022-02023-8 35509081 PMC9066779

[5] Polverino E Goeminne PC McDonnell MJ Aliberti S Marshall SE Loebinger MR . European Respiratory Society guidelines for the management of adult bronchiectasis. Eur Respir J. (2017) 50:1700629. doi: 10.1183/13993003.00629-2017 28889110

[6] Chalmers JD Haworth CS Flume P Long MB Burgel PR Dimakou K . European Respiratory Society clinical practice guideline for the management of adult bronchiectasis. Eur Respir J. (2025) 66:2501126. doi: 10.1183/13993003.01126-2025 41016738

[7] Hill AT Sullivan AL Chalmers JD De Soyza A Elborn JS Floto RA . British Thoracic Society Guideline for bronchiectasis in adults. Thorax. (2019) 74:1–69. doi: 10.1136/thoraxjnl-2018-212463 30545985

[8] Hill AT Haworth CS Aliberti S Barker A Blasi F Boersma W . Pulmonary exacerbation in adults with bronchiectasis: a consensus definition for clinical research. Eur Respir J. (2017) 49:1700051. doi: 10.1183/13993003.00051-2017 28596426

[9] Bhalla M Turcios N Aponte V Jenkins M Leitman BS McCauley DI . Cystic fibrosis: scoring system with thin-section CT. Radiology. (1991) 179:783–8. doi: 10.1148/radiology.179.3.2027992 2027992

[10] Bedi P Chalmers JD Goeminne PC Mai C Saravanamuthu P Velu PP . The BRICS (Bronchiectasis Radiologically Indexed CT Score): a multicenter study score for use in idiopathic and postinfective bronchiectasis. Chest. (2018) 153:1177–86. doi: 10.1016/j.chest.2017.11.033 29247616

[11] Díaz AA Nardelli P Wang W San José Estépar R Yen A Kligerman S . Artificial intelligence-based CT assessment of bronchiectasis: the COPDGene study. Radiology. (2023) 307:e221109. doi: 10.1148/radiol.221109 36511808 PMC10068886

[12] Mayerhoefer ME Materka A Langs G Häggström I Szczypiński P Gibbs P . Introduction to radiomics. J Nucl Med. (2020) 61:488–95. doi: 10.2967/jnumed.118.222893 32060219 PMC9374044

[13] Capaldi DPI Sheikh K Guo F Svenningsen S Etemad-Rezai R Coxson HO . Free-breathing pulmonary 1H and hyperpolarized 3He MRI: comparison in COPD and bronchiectasis. Acad Radiol. (2015) 22:320–9. doi: 10.1016/j.acra.2014.10.003 25491735

[14] Chalmers JD Moffitt KL Suarez-Cuartin G Sibila O Finch S Furrie E . Neutrophil elastase activity is associated with exacerbations and lung function decline in bronchiectasis. Am J Respir Crit Care Med. (2017) 195:1384–93. doi: 10.1164/rccm.201605-1027OC 27911604 PMC5443898

[15] Shoemark A Shteinberg M De Soyza A Haworth CS Richardson H Gao Y . Characterization of eosinophilic bronchiectasis: a European multicohort study. Am J Respir Crit Care Med. (2022) 205:894–902. doi: 10.1164/rccm.202108-1889OC 35050830

[16] Kwok WC Ho JCM Ma TF Lam DC Chan JW Ip M . Risk of hospitalised bronchiectasis exacerbation based on blood eosinophil counts. Int J Tuberc Lung Dis. (2023) 27:61–5. doi: 10.5588/ijtld.22.0489 36853123

[17] De Angelis A Marrades P Perea L Faner R Iorfida A Aliberti S . The importance of symptoms in bronchiectasis. Eur Respir Rev. (2026) 35:250085. doi: 10.1183/16000617.0085-2026 PMC13291825 42342265

[18] Tunney MM Einarsson GG Wei L Drain M Klem ER Cardwell C . Lung microbiota and bacterial abundance in patients with bronchiectasis when clinically stable and during exacerbation. Am J Respir Crit Care Med. (2013) 187:1118–26. doi: 10.1164/rccm.201210-1937OC 23348972 PMC3734618

[19] Rogers GB Zain NM Bruce KD Burr LD Chen AC Rivett DW . A novel microbiota stratification system predicts future exacerbations in bronchiectasis. Ann Am Thorac Soc. (2014) 11:496–503. doi: 10.1513/AnnalsATS.201310-335OC 24592925

[20] Dicker AJ Lonergan M Keir HR Smith AH Pollock J Finch S . The sputum microbiome and clinical outcomes in patients with bronchiectasis: a prospective observational study. Lancet Respir Med. (2021) 9:885–96. doi: 10.1016/S2213-2600(20)30557-9 33961805

[21] Chai YH Xu JF. How does *Pseudomonas aeruginosa* affect the progression of bronchiectasis? Clin Microbiol Infect. (2020) 26:313–8. doi: 10.1016/j.cmi.2019.07.010 31306794

[22] Gu W Miller S Chiu CY. Clinical metagenomic next-generation sequencing for pathogen detection. Annu Rev Pathol. (2019) 14:319–38. doi: 10.1146/annurev-pathmechdis-012418-012751 30355154 PMC6345613

[23] Zhang H Shen D Zhou J Yang Q Ying Y Li N . The utility of metagenomic next-generation sequencing (mNGS) in the management of patients with bronchiectasis: a single-center retrospective study of 93 cases. Open Forum Infect Dis. (2023) 10:ofad425. doi: 10.1093/ofid/ofad425 37663088 PMC10470666

[24] He Z Kong J Su Q Lin J. Application value of metagenomic next-generation sequencing of bronchoalveolar lavage fluid in pathogen detection and diagnostic efficiency of acute exacerbation of bronchiectasis. Am J Transl Res. (2025) 17:4912–25. doi: 10.62347/WOLQ1347 40821031 PMC12351565

[25] van Belkum A Dunne WM Jr. Next-generation antimicrobial susceptibility testing. J Clin Microbiol. (2013) 51:2018–24. doi: 10.1128/JCM.00313-13 23486706 PMC3697721

[26] Aksamit TR O'Donnell AE Barker A Olivier KN Winthrop KL Daniels ML . Adult patients with bronchiectasis: a first look at the US Bronchiectasis Research Registry. Chest. (2017) 151:982–92. doi: 10.1016/j.chest.2016.10.055 27889361 PMC6026266

[27] Pollock J Goeminne PC Aliberti S Polverino E Crichton ML Ringshausen FC . *Aspergillus* serologic findings and clinical outcomes in patients with bronchiectasis: data from the European Bronchiectasis Registry. Chest. (2025) 167:975–92. doi: 10.1016/j.chest.2024.06.3843 39461553

[28] Agusti A Bel E Thomas M Vogelmeier C Brusselle G Holgate S . Treatable traits: toward precision medicine of chronic airway diseases. Eur Respir J. (2016) 47:410–9. doi: 10.1183/13993003.01359-2015 26828055

[29] Marrades P De Angelis A Iorfida A Perea L Voza A Aliberti S . Bronchiectasis and treatable traits: the journey from concept to clinical practice. Respir Med. (2026) 251:108593. doi: 10.1016/j.rmed.2025.108593 41421559

[30] Lee AL Burge AT Holland AE. Airway clearance techniques for bronchiectasis. Cochrane Database Syst Rev. (2015). 2015:CD008351. doi: 10.1002/14651858.CD008351.pub3 PMC7175838 26591003

[31] Spinou A Herrero-Cortina B Aliberti S Goeminne PC Polverino E Dimakou K . Airway clearance management in people with bronchiectasis: data from the European Bronchiectasis Registry (EMBARC). Eur Respir J. (2024) 63:2301689. doi: 10.1183/13993003.01689-2023 38609097 PMC11154755

[32] Lee AL Hill CJ McDonald CF Holland AE. Pulmonary rehabilitation in individuals with non-cystic fibrosis bronchiectasis: a systematic review. Arch Phys Med Rehabil. (2017) 98:774–82. doi: 10.1016/j.apmr.2016.05.017 27320420

[33] Chalmers JD Haworth CS Metersky ML Loebinger MR Blasi F Sibila O . Phase 2 trial of the DPP-1 inhibitor brensocatib in bronchiectasis. N Engl J Med. (2020) 383:2127–37. doi: 10.1056/NEJMoa2021713 32897034

[34] Chalmers JD Burgel PR Daley CL De Soyza A Haworth CS Mauger D . Phase 3 trial of the DPP-1 inhibitor brensocatib in bronchiectasis. N Engl J Med. (2025) 392:1569–81. doi: 10.1056/NEJMoa2411664 40267423

[35] U.S. Food and Drug Administration. Brinsupri (Brensocatib) Tablets, for Oral Use: Prescribing Information. Silver Spring, MD: U.S. Food and Drug Administration (2025).

[36] Chalmers JD Shteinberg M Mall MA O'Donnell AE Watz H Gupta A . Cathepsin C (dipeptidyl peptidase 1) inhibition in adults with bronchiectasis: AIRLEAF, a phase 2 randomised, double-blind, placebo-controlled, dose-finding study. Eur Respir J. (2025) 65:2401551. doi: 10.1183/13993003.01551-2024 39255990 PMC11694546

[37] Congrete S Metersky ML. Telemedicine and remote monitoring as an adjunct to medical management of bronchiectasis. Life. (2021) 11:1196. doi: 10.3390/life11111196 34833072 PMC8622988

[38] Hull RC Stobo J Abo-Leyah H Richardson H Alferes de Lima Headley D Long MB . Endotypes of pseudomonas aeruginosa infection in bronchiectasis are associated with inhaled antibiotic response: results from ORBIT-3 and ORBIT-4. Am J Respir Crit Care Med. (2025) 211:1397–408. doi: 10.1164/rccm.202501-0159OC 40664229 PMC12369955

[39] Chalmers JD Cipolla D Thompson B Davis AM O'Donnell A Tino G . Changes in respiratory symptoms during 48-week treatment with ARD-3150 (inhaled liposomal ciprofloxacin) in bronchiectasis: results from the ORBIT-3 and−4 studies. Eur Respir J. (2020) 56:2000110. doi: 10.1183/13993003.00110-2020 32554534

[40] Haworth CS Shteinberg M Winthrop K Barker A Blasi F Dimakou K . Inhaled colistimethate sodium in patients with bronchiectasis and *Pseudomonas aeruginosa* infection: results of PROMIS-I and PROMIS-II. Lancet Respir Med. (2024) 12:787–98. doi: 10.1016/S2213-2600(24)00225-X 39270696

[41] Chandrasekaran R Mac Aogain M Chalmers JD Elborn SJ Chotirmall SH. Geographic variation in the aetiology, epidemiology and microbiology of bronchiectasis. BMC Pulm Med. (2018) 18:85. doi: 10.1186/s12890-018-0638-0 29788932 PMC5964678

[42] Gao YH Lu HW Mao B Guan WJ Song YL Li YY . The establishment of China Bronchiectasis Registry and Research Collaboration (BE-China): protocol of a prospective multicenter observational study. Respir Res. (2022) 23:328. doi: 10.1186/s12931-022-02254-9 36463140 PMC9719665

[43] Xu JF Zheng HZ Lu HW Wang LW Wu B Lv XD . Baseline characteristics of patients in the Chinese Bronchiectasis Registry (BE-China): a multicentre prospective cohort study. Lancet Respir Med. (2025) 13:166–76. doi: 10.1016/S2213-2600(24)00364-3 39805296

[44] Dhar R Singh S Talwar D Mohan M Tripathi SK Swarnakar R . Bronchiectasis in India: results from the European Multicentre Bronchiectasis Audit and Research Collaboration (EMBARC) and respiratory research network of India registry. Lancet Glob Health. (2019) 7:e1263–72. doi: 10.1016/S2214-109X(19)30327-4 31402007

[45] Dhar R Singh S Talwar D Murali Mohan BV Tripathi SK Swarnakar R . Clinical outcomes of bronchiectasis in India: data from the EMBARC/Respiratory Research Network of India registry. Eur Respir J. (2023) 61:2200611. doi: 10.1183/13993003.00611-2022 36229049 PMC9816417

[46] Chalmers JD Polverino E Crichton ML Ringshausen FC De Soyza A Vendrell M . Bronchiectasis in Europe: data on disease characteristics from the European Bronchiectasis registry (EMBARC). Lancet Respir Med. (2023) 11:637–49. doi: 10.1016/S2213-2600(23)00093-0 37105206

[47] Polverino E Dimakou K Traversi L Bossios A Haworth CS Loebinger MR . Bronchiectasis and asthma: data from the European Bronchiectasis Registry (EMBARC). J Allergy Clin Immunol. (2024) 153:1553–62. doi: 10.1016/j.jaci.2024.01.027 38401857

